# 773. The Role of Infectious Diseases Physicians in Postgraduate Antimicrobial Stewardship Education: A Nationwide Questionnaire Survey in Japan

**DOI:** 10.1093/ofid/ofad500.834

**Published:** 2023-11-27

**Authors:** Toshiki Miwa, Koh Okamoto, Koh Okamoto, Yuji Nishizaki, Yasuharu Tokuda

**Affiliations:** University of Tokyo Hospital, Tokyo, Tokyo, Japan; The University of Tokyo Hospital, Bunkyo, Tokyo, Japan; The University of Tokyo Hospital, Bunkyo, Tokyo, Japan; 2. Division of Medical Education, Juntendo University School of Medicine, Tokyo, Tokyo, Japan; Muribushi Project for Teaching Hospitals, Urasoe, Okinawa, Japan

## Abstract

**Background:**

To promote appropriate antimicrobial use, resident physicians’ understanding of antimicrobial stewardship (AS) is essential. While infectious disease (ID) physicians are expected to play a leading role in AS education, data are limited for the impact of ID physicians’ involvement. We aimed to evaluate the impact of direct interaction with ID physicians on resident physicians’ knowledge, perceptions, and attitudes regarding AS.

**Methods:**

A cross sectional study involving the participants of the Japanese nationwide online examination for resident physicians in January 2023 was performed. The questionnaire with 29 items on their educational background as well as perceptions and attitudes regarding AS were distributed, collected, and analyzed in combination with the examination scores.

**Results:**

Of 8,438 post-graduate year (PGY) 1 and 2 resident physicians participated in the examination, 4,051 (48.0%) from 577 institutions responded in the present study. A half (54.0%) worked at hospitals with an existing ID department (Table 1). A third (32.7%) did not receive any formal AS education during their residency program whereas 65.2% failed to understand the concept of AS, and 79.2% were less confident in choosing appropriate antimicrobials. Among respondents working at hospitals with an ID department, presence of bedside ID consultation service was associated with enhanced commitment to choosing narrow-spectrum antimicrobials, de-escalation, transition to oral therapy, and compliance of appropriate duration of therapy than ID consultation only based on chart review (49.6% vs. 42.6%, 51.4% vs. 41.6%, 76.3% vs. 66.3%, 66.7% vs. 55.7%, respectively [all of *P* < 0.05]) (Table 2). Although there was no statistically significant difference in total test scores, respondents who had an ID rotation were more likely to review past antimicrobial exposure and colonization of resistant pathogens and try to choose narrow-spectrum antimicrobials than those who received lectures only (59.3% vs 39.0%, *P* < 0.001) (Figure).

**Figure:**
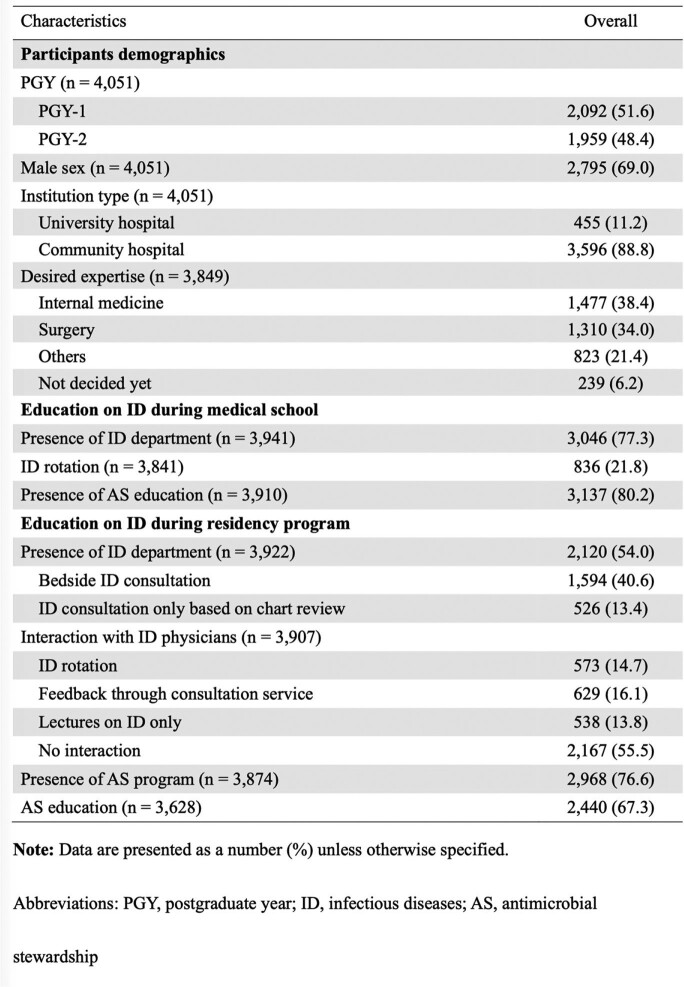

**Figure:**
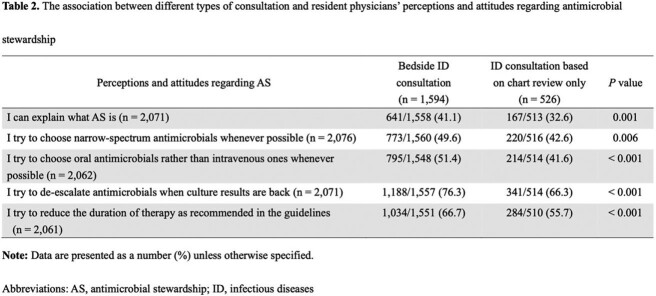

**Figure:**
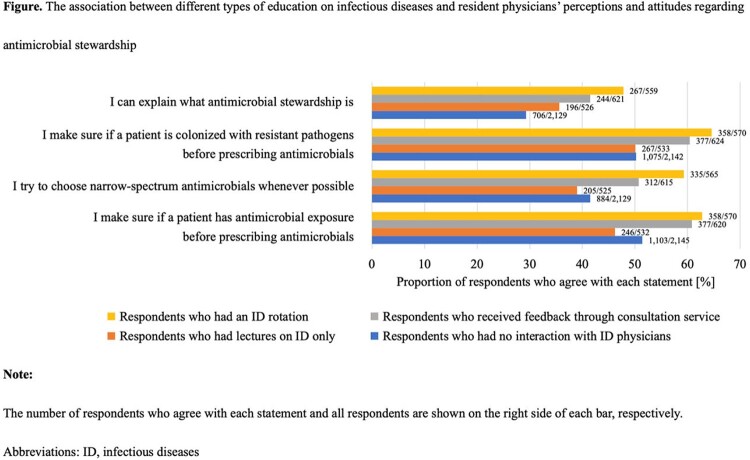

**Conclusion:**

Resident physicians’ perceptions and attitudes regarding AS may improve through direct interaction with ID physicians. Our findings underscore the imperative role and value of ID physicians as key educators for future physicians.

**Disclosures:**

**All Authors**: No reported disclosures

